# Advances in Wood Composites III

**DOI:** 10.3390/polym13010163

**Published:** 2021-01-05

**Authors:** Antonios N. Papadopoulos

**Affiliations:** Laboratory of Wood Chemistry and Technology, Department of Forestry and Natural Environment, International Hellenic University, GR-661 00 Drama, Greece; antpap@for.ihu.gr

Wood composites are man-made materials that can be easily manufactured from a variety of raw lignocellulosic materials and the appropriate binder [1]. They are advantageous because they can be successfully designed for specific end-uses and appropriate performance requirements, which enables them to be used in a variety of applications without sacrificing the final structural requirements. In addition, wood composites are able to be tailored to a wide array of applications, a fact that can contribute to the reduction of the use three types of solid wood. This particular Special Issue, “Advances in Wood Composites III”, is a continuation of two previous successful series, namely, “Advances in Wood Composites I” and “Advances in Wood Composites II” [2,3] and gives a presentation of recent progress and development in refining and enhancing the overall performance of wood composites. The Special Issue, with a collection of 14 original, high-quality research papers, provides selected examples of recent Advances in Wood Composites.

In this Special Issue, a great deal of attention was given to the use of nanomaterials, as an alternative way to improve basic properties of wood composites. Gul et al. [4] examined the effect of iron oxide nanoparticles, at three loadings, upon the physical properties of laboratory-made medium-density fiberboard from poplar fibers. The nanoparticles were firstly dispersed into commercial urea formaldehyde resin and then were sprayed onto fibers. The results revealed that the incorporation of the iron oxide particles improved key physical properties, such as thickness swelling and water absorption. Furthermore, it was stated that the heat transfer and the curing of the resin was significantly improved as a result of the higher reactivity and the higher surface area of the nanoparticles [4,5].

In another paper, a wood-SiO_2_ composite material was manufactured using nano silica sol. This was introduced into Chinese fir via in-situ polymerization applying vacuum impregnation [6]. It was found that nano silica can effectively penetrate into the cell cavities and to the surface of cell walls and therefore combine with the substances of wood. The developed method improved the compact microstructure and further improved mechanical and thermal properties. The hydrophobicity of the composite material was also improved. It seems that nanomaterials, as extensively presented and discussed in the previous Special Issues, improve the properties of wood as raw material and alter its original feature to a limited extent; potential changes in wood properties are based in the higher interfacial area which is developed due to treatment.

Research on the utilization of nanofibers from cellulose has been widely reported. Huang and co-workers [7] studied the effect of nanoclay incorporation upon the performance of cellulose nanofiber film. It was found that films with low nanoclay content showed better mechanical performance and, when exposed to an open flame, demonstrated a self-extinguishing ability. In addition, it was reported that the incorporation of montmorillonite improved the hydrophobicity of the material. It was concluded that this materials can be used as a barrier for packaging applications.

Wang et al. [8], made spherical cellulose nanofiber aerogels via two drying techniques, namely, liquid nitrogen freeze drying and supercritical CO_2_ drying, and their chemical and physical properties were studied. It was reported that both methods resulted at similar structures of the aerogels. However, the aerogels made liquid nitrogen freeze drying, exhibited higher pore volume, lower surface area, higher density and excellent shrinkage.

Lignocellulose plastic composites are a combination of processing techniques for filler and fiber preparation and polymer science [9,10]. Wang and co-workers manufacture a lignocelluloses plastic composite using polylactic acid and bamboo charcoal as a filler for bamboo powder. As a fire retardant, aluminum hypophosphite was added. It was found that the bamboo charcoal had a detrimental effect on flexural properties and the addition of the flame retardant enhanced the bonding between polylactic acid acid and bamboo charcoal. Overall, it was concluded that the formation of carbonized surface layers in the produced composite reduced the fire growth index and improved the fire performance index. In the same context, a lignin-based flame retardant was manufactured in order to improve thermal properties of epoxy resin due to its poor flammability [11].

Bark panels have recently received some attention and the performance of composites made of various bark species were investigated by Kain et al. [12]. Insulation panels were made and a variety of parameters were examined, such as bark species, resin content, resin type and density. It was found that the bark species had a very minor effect on the properties of the panel. It was concluded that the thermal conductivity of the insulation panels is predominantly affected by the density of the panel. In another approach, bark from larch was used as a formaldehyde scavenger in insulation panels, manufactured with three different resins, namely, melamine-urea formaldehyde, urea formaldehyde and a tannin-based adhesive [13]. It was reported that all panels belonged to the super E0 classification for free formaldehyde content and recommended this raw material for further research in green construction solutions.

The main drawbacks of wood, namely, susceptibility to biodegradability and dimensional instability, can be addressed by chemical or thermal modification and inorganic modification by sol-gel technologies. Among the sol-gel technologies, the sol-gel derived wood-inorganic composite (WIC) approaches have received a lot of attention over the last few years.

An interesting attempt was made to examine the performance of a casein-based adhesive in manufacturing biodegradable ski cores made from ash veneers [14]. A variety of combinations containing casein powder, sodium silicate, water, lime and resin content were applied, and the final composite, three-layer plywood, was tested for mechanical and physical properties. Traditional wood adhesives such as polyvinyl acetate and epoxy were used as controls. Significant differences were reported between the various combinations, leading to the conclusion that there is an affinity between the ratio of the components, glue amount and physical and mechanical plywood performance. The highest efficiency was reported for the plywood glued with casein and increased quantity of lime.

High strength plywood from partially delignified densified spruce wood was made by Jakob et al. [15]. Delignified and densified veneers were prepared by means of alkaline and organosolv approach. Plywood was made using phenol-resorcinol-formaldehyde adhesive. Bending properties were significantly improved, but interlaminar shear strength values were not so promising. It was concluded that the plywood from partially delignified densified veneers can be characterized as promising and shows a potential to compete with woven fiber reinforrced polymers.

Another interesting topic that was addressed in this issue is the transparency of wood. Lignin was gradually removed from poplar veneers and subsequently epoxy resin was filled into them [16]. The results showed that the cell wall became thin and the porosity of the veneers was significantly augmented with the removal of lignin. In addition, the light transmittance was increased and the thermal stability became more stable. It is concluded that this material has the potential to be used as a substrate in solar cells, smart windows and photovoltaics.

Policardi and Thebault [17] evaluated the buffer capacities of eight wood species in particleboard production. It was found that the buffer capacity of oak wood was rather extreme and concluded that it is possible to affect the ability of both melamine-urea formaldehyde and pure formaldehyde resin in industrial operation. Furthermore, it is highlighted that the above-mentioned resins are negatively affected by such a strong oak wood furnish buffer capacity.

Last but not least, the issue of volatile organic compounds (VOCs) from wood and wood composites is addressed [18]. Adamova and co-workers presented a comprehensive review on methods of evaluation, mitigation and potential health risks.

It can be safely and definitely said that this field, the field of wood composites, is a fascinating one and its future looks more than bright. What is presented herein and in the other two Editorials is just a very short overview of what will happen in the near future. Laboratories worldwide do innovative research, and new challenges, approaches and ideas are continuously increasing, allowing us to mirror an exciting and interesting research future. So, after the three successful Special Issues in the field “Advances in Wood Composites”, a forth one is now available and open for submission.

## References

[1] Pizzi A., Papadopoulos A.N., Policardi F. (2020). Wood composites and their polymer binders. Polymers.

[2] Papadopoulos A.N. (2020). Advances in Wood Composites. Polymers.

[3] Papadopoulos A.N. (2020). Advances in Wood Composites II. Polymers.

[4] Gul W., Alrobei H., Shah S.R.A., Khan A. (2020). Effect of Iron Oxide Nanoparticles on the Physical Properties of Medium Density Fiberboard. Polymers.

[5] Papadopoulos A.N., Taghiyari H.R. (2019). Innovative wood surface treatments based on nanotechnology. Coatings.

[6] Xu E., Zhang Y., Lin L. (2020). Improvement of Mechanical, Hydrophobicity and Thermal Properties of Chinese Fir Wood by Impregnation of Nano Silica Sol. Polymers.

[7] Huang R., Zhang X., Li H., Zhou D., Wu Q. (2020). Bio-Composites Consisting of Cellulose Nanofibers and Na^+^ Montmorillonite Clay: Morphology and Performance Property. Polymers.

[8] Wang Z., Zhu W., Huang R., Zhang Y., Jia C., Zhao H., Chen W., Xue Y. (2020). Fabrication and Characterization of Cellulose Nanofiber Aerogels Prepared via Two Different Drying Techniques. Polymers.

[9] Wang S., Zhang L., Semple K., Zhang M., Zhang W., Dai C. (2020). Development of Biodegradable Flame-Retardant Bamboo Charcoal Composites, Part I: Thermal and Elemental Analyses. Polymers.

[10] Wang S., Zhang L., Semple K., Zhang M., Zhang W., Dai C. (2020). Development of Biodegradable Flame-Retardant Bamboo Charcoal Composites, Part II: Thermal Degradation, Gas Phase, and Elemental Analyses. Polymers.

[11] Liang X., Hu Q., Wang X., Li L., Dong Y., Sun C., Hu C., Gu X. (2020). Thermal Kinetics of a Lignin-Based Flame Retardant. Polymers.

[12] Kain G., Tudor E.M., Barbu M.-C. (2020). Bark Thermal Insulation Panels: An Explorative Study on the Effects of Bark Species. Polymers.

[13] Barbu M.C., Lohninger Y., Hofmann S., Kain G., Petutschnigg A., Tudor E.M. (2020). Larch Bark as a Formaldehyde Scavenger in Thermal Insulation Panels. Polymers.

[14] Schwarzenbrunner R., Barbu M.C., Petutschnigg A., Tudor E.M. (2020). Water-Resistant Casein-Based Adhesives for Veneer Bonding in Biodegradable Ski Cores. Polymers.

[15] Jakob M., Stemmer G., Czabany I., Müller U., Gindl-Altmutter W. (2020). Preparation of High Strength Plywood from Partially Delignified Densified Wood. Polymers.

[16] Lu M., He W., Li Z., Qiang H., Cao J., Guo F., Wang R., Guo Z. (2020). Effect of Lignin Content on Properties of Flexible Transparent Poplar Veneer Fabricated by Impregnation with Epoxy Resin. Polymers.

[17] Policardi F., Thebault M. (2020). The Buffer Effect of Different Wood Species and the Influence of Oak on Panel Composites Binders. Polymers.

[18] Adamová T., Hradecký J., Pánek M. (2020). Volatile Organic Compounds (VOCs) from Wood and Wood-Based Panels: Methods for Evaluation, Potential Health Risks, and Mitigation. Polymers.

